# Differentiation of Subclinical Ketosis and Liver Function Test Indices in Adipose Tissues Associated With Hyperketonemia in Postpartum Dairy Cattle

**DOI:** 10.3389/fvets.2021.796494

**Published:** 2022-02-03

**Authors:** Muhammad Ali Mohsin, Huiru Yu, Rongze He, Peng Wang, Linli Gan, Yulan Du, Yunfei Huang, Muhammad Bakhsh Abro, Sarmad Sohaib, Mariusz Pierzchala, Przemysław Sobiech, Klaudia Miętkiewska, Chandra S. Pareek, Bao Xiang He

**Affiliations:** ^1^Laboratory of Clinical Veterinary Medicine, College of Animal Science and Technology, Guangxi University, Nanning, China; ^2^Shanghai Animal Disease Prevention and Control Center, Shanghai, China; ^3^School of Life Science and Engineering, Foshan University, Guangdong, China; ^4^Department of Veterinary Medicine, Faculty of Veterinary and Animal Science, Lasbela University of Agriculture, Water, and Marine Sciences, Uthal, Pakistan; ^5^Department of Genomics and Biodiversity, Institute of Genetics and Animal Biotechnology of the Polish Academy of Sciences, Jastrzebiec, Poland; ^6^Internal Disease Unit, Department of Clinical Sciences, Faculty of Veterinary Medicine, University of Warmia and Mazury in Olsztyn, Olsztyn, Poland; ^7^Department of Basic and Preclinical Sciences, Institute of Veterinary Medicine, Faculty of Biological and Veterinary Sciences, Nicolaus Copernicus University, Torun, Poland; ^8^Division of Functional Genomics in Biological and Biomedical Research, Centre for Modern Interdisciplinary Technologies, Nicolaus Copernicus University, Torun, Poland

**Keywords:** dairy cow, subclinical ketosis, hyperketonemia, growth hormone, adipose tissue

## Abstract

Past studies suggested that during early lactation and the transition period, higher plasma growth hormone (GH) levels in subclinical ketosis (SCK) might involve the initiation of body adipose tissues mobilization, resulting in metabolic disorders in ruminants particularly hyperketonemia. The upregulated GH mRNA expression in adipose tissue may take part in the adipolysis process in SCK-affected cows that paves a way for study further. This study aimed to characterize the plasma levels of GH, β-hydroxybutyrate acid (BHBA) and non-esterified fatty acid (NEFA) and glucose (GLu) in ketotic cows and healthy control (CON) cows; to measure the liver function test (LFT) indices in ketotic and healthy CON cows, and finally the quantitative real-time PCR (qRT-PCR) assay of candidate genes expressed in adipose tissues of ketotic and healthy CON cows during 0 to 7 week postpartum. Three experiments were conducted. Experiment-1 involved 21 Holstein cows weighing 500–600 kg with 2–5 parities. Results showed that GH, BHBA, and NEFA levels in ketotic cows were significantly higher and the GLu level significantly lower. Pearson's correlation analysis revealed a significant positive correlation of GH with BHBA, NEFA, and GLu in ketotic and healthy CON cows. In experiment-2, dynamic monitoring of LFT indices namely, alanine aminotransferase (ALT), aspartate aminotransferase (AST), γ-glutamyl transpeptidase (GGT), total bilirubin (TBIL), direct bilirubin (DBIL), total protein (TP), albumin (ALB), globulin (GLOB) and albumin/globulin (A/G) were examined. The TBIL, DBIL, and GGT indices were significantly higher in ketotic cows and TP was significantly lower. In experiment-3, mRNA expression levels of GHR and peroxisome-proliferator-activated receptor alpha (PPARα) genes in adipose tissue were significantly upregulated in ketotic cows. However, the mRNA expression of insulin-like growth factor-I (IGF-1), insulin-like growth factor-I receptor (IGF-1R), and sterol regulatory element-binding protein-1c (SREBP-1c) genes in adipose tissue were downregulated in ketotic cows. Our study concluded that during postpartum, higher plasma GH levels in SCK cows might involve the initiation of body adipose tissue mobilization, resulting in hyperketonemia.

## Introduction

Growth hormone (GH) is a naturally occurring peptide hormone produced by the pituitary gland that promotes growth and metabolism (1). Growth hormone receptors (GHRs) mediate the actions of GH by binding GHRs and transducing an intracellular signal through the activation of signal transducer and activator of transcription 5 (STAT5) (2). In the liver, GH causes the synthesis and secretion of insulin-like growth factor-I (IGF-I) via a series of terminal Stat 5 binding sites of the IGF-I gene. Earlier reports suggested that nutrient partitioning and other metabolic activities are the essential physiological processes that subsequently help in subclinical ketosis (SCK) prevention (3) and improving cow's health during the transition and early lactation periods of dairy cattle. In general, the fatty acids, blood metabolites and GH directly or indirectly influence animals in terms of growth, reproduction, welfare condition, during the transition and early lactation periods (4–7). During the early lactation period, dairy cows experience tremendous physiological and metabolic changes (8–10), accompanied by fetal development, milk production, high energy intake and a negative energy balance (NEB). Dairy cows experience an NEB due to reduced dry matter intake, while higher energy production is utilized for milk production (11, 12). Severe NEB initiates fat mobilization (13), metabolic stress (14, 15) and subsequently increases the blood concentrations of fatty acids and β-hydroxybutyrate (BHB), which can result in hyperketonemia development (16). The most prevalent metabolic characteristics of hyperketonemia are the increased plasma concentration of the ketone bodies, including acetone, acetoacetate and BHB. Commonly, at 4–8 weeks after calving, Holstein dairy cows accomplish a lactation peak with potential metabolites alterations and require a balanced energy diet [(17): Frontier]. However, insufficient food intake and the physiological changes after calving lower the appetite, which does not recover quickly (appetite peak can be attained 10–12 weeks after birth). The energy obtained from the animal's body through dry matter (DM) absorption cannot meet the body's requirements, which are significant during lactation (7, 18). Therefore, the animal's body first mobilizes hepatic glycogen and then fats and proteins to accelerate gluconeogenesis to maintain the energy balance needed for lactation. The higher incidence of SCK in sheep is also meticulously interrelated with hepatic injuries, hormones and biochemical entity variations (19, 20). It has been reported that GH injections have produced hyperketonemia in early lactating cows (18), which can stimulate lipolysis, possibly by increasing lipolysis and increasing free fatty acid (FFA) concentration (21–23). The increased FFA concentration can lead to enhanced hepatic ketogenesis (23). Some researchers have shown that downregulation of GHR in dairy cows elevated the GH level in the blood and reduced the different important factors, including IGF during the early lactation period (24, 25). Thus, these studies demonstrated that the GH–IGF-I axis is uncoupled in early lactating cows. A harsh summer season, together with heat and humidity, creates a very uncomfortable environment and leads to substantial economic losses through its detrimental effect on cow rumen health, metabolism, production and reproduction (26). The nutrients of the peripheral tissues are preferentially given to the mammary glands. Hyperketonemia is a complex nutrient metabolic disease in postpartum dairy cows. The negative balance of energy metabolism leads to excessive body fat mobilization, which triggers excessive production of ketone bodies in the liver resulting in an abnormal increase in blood levels. The GH protagonism in hyperketonemia etiology remains unclear. Thus, we premised that metabolic regulation retained by GH led to increased milk production, an NEB and hyperketonemia. Therefore, we designed the current study to determine the liver function test (LFT) indices, serum GH concentration levels and its dynamic variations in cows with postpartum SCK, and adipose tissue mRNA expression of the molecules related to the GH regulation pathway to reveal the mechanism of hyperketonemia in cows.

## Materials and Methods

### Ethical Approval

This study was conducted according to the recommendations and guidelines for the care and use of animals, of the College of Animal Science and Technology, Guangxi university under the supervision and protocol approved by the Committee GXU-2017-026.

### Animals and Experimental Design

In this study, we conducted three experiments. The experimental animals were selected during the last stage of the peripartum period of Holstein dairy cattle reared at the Guangxi University Jinguang experimental dairy farm at Nanning, Guangxi Zhuang Autonomous Region, China.

**Figure 1:**
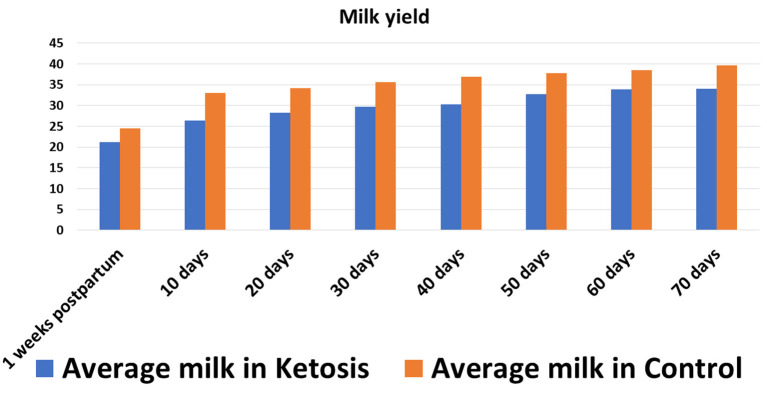
Average milk yield in SCK and CON healthy dairy cows during 0 to 70 days postpartum.

In experiment 1, the SCK experimental trial was conducted by monitoring the milk performance records of 37 Holstein dairy cows. The investigated Holstein dairy cows weighed, on average, 533.14 ± 5.83 kg, with an average milk yield of 31 ± 5 L (Figure 1), a body condition score (BCS) of 3.23 ± 0.06 and 2–5 parities. During the experimental trial, blood samples (*n* = 37) were collected eight times in total, once a week peripartum and from the day of delivery (0 weeks) to seven weeks peripartum, to determine the plasma levels of GH and ketosis-related β-hydroxybutyric acid (BHBA), non-esterified fatty acid (NEFA) and glucose (GLu) indices (experiment 1). Negative energy balance often occurs within 7 weeks postpartum in high-yielding cattle, which causes body fat mobilization and ketosis in severe cases. In this experiment, blood samples were collected week by week within 7 weeks after delivery to dynamically monitor the negative energy balance and the occurrence of ketosis, as well as the relationship between ketosis bodies and other indicators, so as to provide a basis for revealing the occurrence mechanism of ketosis. After completion of the blood plasma measurements, 16 Holstein cows with comorbidities were culled and a total of 21 Holstein cows qualified and selected for further investigation. According to the diagnostic criteria of SCK (BHBA ≥ 1.2 mmol/L without clinical symptoms), a total of 11 Holstein cows were assigned to the SCK group and 10 healthy Holstein cows to the control (CON) group (17).

In experiment 2, the qualified Holstein cows (*n* = 21) were further investigated to determine the LFT of SCK affected cows during the postpartum period. The dynamic monitoring of LFT indices namely, alanine aminotransferase (ALT), aspartate aminotransferase (AST), γ-glutamyl transpeptidase (GGT), total bilirubin (TBIL), direct bilirubin (DBIL), total protein (TP), albumin (ALB), globulin (GLOB) and albumin/globulin (A/G) was carried out on 11 ketotic cows and 10 healthy cows of the CON group for 2 months starting from the day of delivery at 0 weeks to the seventh week postpartum period, to identify the changes of LFT indices in the two groups as well as to determine the correlation between the GH-related factors of SCK and LFT indices.

In experiment 3, gene expression analysis in adipose tissue was performed on 10 peripartum Holstein cows after the calving period. The purpose of this was to validate the variations of the gene expression levels in the GH–IGF-1 axis of fat tissues in SCK affected Holstein cows. The subcutaneous adipose tissues of Holstein cows were collected by a surgical biopsy to detect mRNA expression levels of GH-related factors in SCK and healthy dairy cows, and to explore the mechanism of GH in lipid metabolism and the relationship with hyperketonemia. Five Holstein cows diagnosed with SCK by the ketone powder method and BHBA test were selected as the SCK group, and paired according to parity, calving date and milk yield. Five healthy cows were selected as the CON group. The subcutaneous adipose tissue of the cow's tail was harvested surgically and the mRNA expression of growth hormone-related signaling factors namely Peroxisome-proliferator-activated receptor alpha (PPARα) and sterol regulatory element-binding protein-1c (SREBP-1c), GHR, IGF-1, IGF-1R, STAT5, serine/threonine protein kinase B (AKT), were determined among the healthy and SCK affected Holstein cows. The postoperative care for animals after surgery was performed as follows. After animal surgery, small incisions (about 1 cm) were sutured, and the incision was disinfected with iodophor and wrapped with sterile gauze. Gauze was removed after 2-3 days, and the wound was observed for infection, and disinfected with iodophor every day. The wound was cured in 5-7 days.

### Experimental Feeding of Holstein Cows During the Peripartum (Experiment 1) and Postpartum (Experiment 2 and Experiment 3) Periods

For feeding purposes, two types of rations were formulated as devised by a nutritionist, and the basal diet formulation was (Table 1) according to a previous study by Huang et al. (27). For all investigated animals (*n* = 37), the first ration for the peripartum period and the second ration for the postpartum period were fed ad libitum once per day (Table 1).

**Table 1:** The ingredients and chemical composition of experimental feedstuffs^a^ of diets/rations during the peripartum period of dairy cows.

**Diet composition ingredients (% of DM and nutrient compositions^c^)**	**Proportion ratio%**
Corn in northeast china	24.16
Corn silage	4.35
Wheat bran	3.17
Bean pulp	9.05
Rape seed dregs	5.43
Alfalfa silage	16.83
Soybean hulls	10.76
Elephant grass	3.55
Chinese leymus	8.7
Calcium carbonate	0.51
Calcium hydrophosphate	1.02
Sodium bicarbonate	0.41
Animal salt	0.48
Vitamin mineral premix^b^	0.7
Beer brewing wastes	10.88
**Nutrient content**	**Proportion ratio %**
DM (accounting for feed ratio %)	54.93
Crude protein CP (ratio of DM accounting % DM)	20.32
NDF neutral detergent fiber (representing dry ratio of % DM)	32.02
Acid detergent fiber ADF (representing dry ratio of % DM)	12.77
Cow NE_L_^d^ (Mcal/kg), DM (Mcal/kg of DM)	1.73

a *DM, dry matter; CP, crude protein; NDF, neutral detergent fiber; ADF, acid detergent fiber; NE_L_, net energy lactation*.

b *Content per kilogram of premix: 450-550K IU of vitamin A; 140 to 160K IU of vitamin D_3_; 2,900-3,100IU of vitamin E; 3.9-4.1g of Fe; 1.2-1.4 g of Cu; 2.9-3.1 g of Mn; 5.9-6.1 g of Zn; 75-85 mg of I; 45-55 mg of Se; 75-85 mg of Co*.

c *Calculated from the analyzed value of the feedstuffs ingredients*.

d *NE_L_, Net Energy lactation; calculated based on feedstuffs according to the Chinese National Station of Animal Production and Health (2000)*.

### Selection Criteria of the Grouping of SCK and CON Dairy Cows During the Peripartum Period

The ketosis powder test was used for on-site screening of Holstein cows with positive results (BHBA ≥ 1.2) and were further analyzed for other parameters on the same day. Based on long-term monitoring experience in cattle farms, healthy cows with the same parity and similar production dates were selected as the CON group, if the plasma BHBA concentration was less than 1.2 mmol/l, and there were no clinical symptoms like digestive issues, loss of appetite, sudden weight loss or neurotype of sudden onset, blind or circular movements, tense muscles, trembling and milk fever (28). The cows were assigned to the SCK group if the plasma BHBA concentration was higher than 1.2 mmol/L in any week from the date of delivery at 0 weeks to 7 weeks postpartum. Most cows achieved BHB ≥ 1.2 mmol/L in the first week after calving (29, 30).

### Determination of Plasma Levels of GH, BHBA, NEFA, and GLu (Experiment 1)

In experiment 1, the blood samples (*n* = 21) were collected once a week from 0 weeks to 7 weeks postpartum, and ketosis-related parameters such as GH, BHBA, NEFA, and GLu were measured. Blood samples were collected at 7:00~8:00 am from the jugular vein (30) into 10 ml tubes with heparin sodium as an anticoagulant on days 0, 7, 14, 21, 28, 35, and 42 of the calving dates.

The plasma concentrations of GLu, BHB, and NEFA were determined using a Hitachi 7170 auto-analyzer (Hitachi, Tokyo, Japan) and a commercially available kit [glucose: catalog number GL3815; BHB: catalog number RB1008; (Randox Laboratories, Crumlin, UK)] according to the manufacturer's instructions. Radioimmunoassay (RIA) determined the concentrations of GH in serum and medium. The antibody against bovine GH was purchased from Abcam (Cambridge, UK; ab31496). The intra- and inter-coefficients of variation assays for the GH and IGF-I were <5%. In this study, sample collection times were the same for both groups. Samples were placed in a centrifuge tube with a 1% heparin physiological saline solution of 55 μl, and the mixture inverted. The blood was centrifuged at room temperature for 10 min at 3,500 rpm/min, and the plasma was separated. The plasma was dispensed into 1.5 ml Eppendorf tubes and placed in an icebox and sent to the laboratory. Blood glucose was detected immediately after being returned to the laboratory, and the remaining plasma was stored in a deep refrigerator at −80°C for further testing.

### Determination of LFT in Dairy Cows (Experiment 2)

The LFT indices, namely, alanine aminotransferase (ALT), aspartate aminotransferase (AST), γ-glutamyl transpeptidase (GGT), total bilirubin (TBIL), direct bilirubin (DBIL), total protein (TP), albumin (ALB), globulin (GLOB) and albumin/globulin (A/G) were analyzed using a commercial kit according to the manufacturer's instructions. The ALT test was measured by continuous monitoring method; AST test was measured by continuous monitoring method; GGT test was measured by γ- glutamyl-3-carboxy-4-nitroaniline method; TBIL test was measured by vanadate oxidation; DBIL test was measured by vanadate oxidation; TP test was measured by Biuret; ALB test was measured by Biuret; GLOB test was measured by Bromocresol green method; and A/G test was measured by commercial A/G ratio test (31).

### Quantitative Real-Time PCR Assay of Candidate Genes Expressed in Adipose Tissue of Dairy Cows (Experiment 3)

The mRNA expression of GHR, JAK2-STAT5, IGF-1, IGF-1R, PI3K-AKT, SREBP-1c, and PPARα in ketotic and CON healthy dairy cows were determined in adipose tissue, to understand the mechanism of GH in lipid metabolism and its relationship with hyperketonemia. For this purpose, five experimental cows identified with hyperketonemia by the ketone powder method combined with BHBA quantitative determination were selected as the SCK group (32). Five experimental healthy cows paired with similar conditions based on calving date, parity and milk yield were selected as the CON group. The subcutaneous adipose tissue under the cow's tail was harvested surgically and cut into bean-sized pieces, put in a freezing tube, placed in a liquid nitrogen tank and stored at −80°C until analysis. The mRNA expression of GHR, JAK2-STAT5, IGF-1, IGF-1R, PI3K-AKT, SREBP-1c, and PPARα were determined using qRT-PCR assay (29, 30, 32).

### Laboratory Procedure for qRT-PCR

The total RNA was extracted from subcutaneous adipose tissue using an RNA-plus kit (TaKaRa Biotechnology Co. Ltd., Dalian, China) according to the manufacturer's instructions. The RNA quality and quantity were measured using a nanodrop spectrophotometer and RNA integrity was determined by gel electrophoresis. The total cDNA was reverse-transcribed by using a reverse transcription kit (TaKaRa Biotechnology Co. Ltd.). The relative mRNA expression of target genes was detected using the FastStart Universal SYBR Green Master (ROX) (Roche, Norwalk, CT) on the Roche LightCycler96 real-time PCR system (Roche, Mannheim, Germany). The primers of target genes were designed by primer express software (Applied Biosystems Inc.) using the gene sequences published in GeneBank (Table 2). The β-actin gene was used as an endogenous reference gene (32), and its normalization was performed for both SCK and CON group samples. The reaction conditions were 95°C for 3 min, followed by 40 cycles of 95°C for 15 s and 60°C for 1 min. The relative quantitation values were normalized to the geometric mean of each reference gene's Cq. Quantification cycle values were extrapolated using the 2 – ΔΔCq method (33).

**Table 2:** Sequence of target gene primers utilized in qRT-PCR assay.

**Target gene name**	**GenBank accession no**.	**Primer sequence**	**Amplicon size**
GHR	NM_176608.1	For CCAGTTTCCATGGTTCTTAATTAT Rev TTCCTTTAATCTTTGGAACTGG	138 bp
STAT5A	NM_001012673	For TGTGGAGTTTGAGGTGAAGC Rev ATTATCAAAGAAGGGCTGCAC	113 bp
IGF-1	NM_001012673	For TCGCATCTCTTCTATCTGGCCCTGT Rev GCAGTACATCTCCAGCCTCCTCAGA	101 bp
IGF-1R	NM_001244612.1	For TTAAAATGGCCAGAACCTGAG Rev ATTATAACCAAGCCTCCCAC	240 bp
AKT	NM_173986	For CACGTGCTCTGGACGCTTC Rev ATGGCGAGGTTCCACTCAAAC	314 bp
SREBP-1c	NM_001113302.1	For GCAGCCCATTCATCAGCCAGACC Rev CGACACCACCAGCATCAACCACG	102 bp
PPARα	NM_001034036	For AATAACGCGATTCGTTTTGG Rev TCCATGTCGTGGATGAGAAA	113 bp
β-actin (reference gene)	NM173979	For CTCTTCCAGCCTTCCTTCCT Rev GGGCAGTGATCTCTTTCTGC	233 bp

### Statistical Analysis

Descriptive and inferential statistics for experiment 1 and experiment 2 were calculated using SPSS, version 20.0 for Windows (SPSS Inc., Chicago IL, USA) and independent samples were calculated by the *T*-test. The results were expressed as least-squares mean (LSM) ± standard error (SE). Dynamic monitoring of plasma level of GH, BHBA, NEFA, and GLu indices in SCK and healthy CON dairy cows (longitudinal study) were measured using a mixed effect multiple regression model fitting analysis (JMP10: https://www.jmp.com/en_gb/support.html). The fixed effects of independent variables (groups, number of weeks) were fitted to the statistical model. In this model, the cows were used as random effects. The results of fixed effects (groups and weeks) were compared with the standard LSM ± SE with expressed significant difference indicated in terms of *p*-values. The correlation analysis of plasma levels of GH, BHBA, NEFA, GLu, and LFT indices were carried out using the Pearson correlation coefficient (r), and effects of significance were expressed using *p*-values (34). The experimental data of the qRT-PCR assay (experiment 3) were analyzed using SPSS, version 20.0. (SPSS Inc., Chicago IL, USA). The significant differences between groups were analyzed by ANOVA, and the data were expressed in the form of mean ± SD. The significance of differences expressed in different letters was indicated as *p* < 0.01, *p* < 0.05.

## Results

### Determination of the Plasma Levels of GH, BHBA, NEFA, and GLu Monitored Weekly During 0 to 7 Weeks of Postpartum

The estimates of plasma levels of GH, BHBA, NEFA and GLu during 0 to 7 weeks of postpartum showed that a relatively higher concentration of GH (*p* = 0.0018), BHBA (*p* < 0.0001) and NEFA (*p* = 0.0025) in dairy cows with SCK than the healthy CON group of cows. However, a higher level of GLu was observed in the CON group (*p* = 0.0170) than in the SCK group of dairy cows (Table 3).

**Table 3:** Determination of blood plasma levels of GH, BHBA, NEFA GLu indices in SCK and healthy control cows.

**Group**	**N**	**GH**	**BHBA**	**NEFA**	**GLu**
		**ng/ml**	**mmol/L**	**mmol/L**	**mmol/L**
SCK	11	9.634 ± 3.535	1.894 ± 0.180	1.038 ± 0.125	2.923 ± 0.095
CON	10	3.947 ± 0.242	0.523 ± 0.179	0.452 ± 0.125	3.261 ± 0.095
*p*-value		0.0018	<0.0001	0.0025	0.0170

The correlation coefficient (r) analysis results showed the positive correlations for GH-BHBA and BHBA-NEFA, the negative correlations between BHBA-GLu, and NEFA-GLu, and no correlations for GH-NEFA and GH-GLu in the SCK group of dairy cows (Table 4). Moreover, there was a positive correlation for GH-GLu, the negative correlations for GH-BHBA, GH-NEFA, and BHBA-NEFA, and no correlations for GH-BHBA and GH-NEFA in the CON group of healthy dairy cows (Table 4).

**Table 4:** Correlation coefficient (r) analysis among plasma levels of GH, BHBA, NEFA, and GLu in SCK and CON healthy dairy cows (*n* = 11) monitored weekly eight times during 0 to 7 weeks of postpartum.

**SCK group**	**GH**	**BHBA**	**NEFA**	**GLu**
GH	-	0.256^**^	0.034	0.091
BHBA	0.16	-	0.212^**^	−0.382^**^
NEFA	0.755	0.047	-	−0.209^*^
GLu	0.402	0.000	0.048	-
**CON group**	**GH**	**BHBA**	**NEFA**	**GLu**
GH	-	−0.41	−0.282^*^	0.384^**^
BHBA	0.718	-	−0.176	0.059
NEFA	0.011	0.119	-	0.089
GLu	0.000	0.604	0.434	-

^*^Indicates that the correlation of p < 0.05 is significant and ^**^indicates that the correlation of p < 0.01 is highly significant; the upper right corner is the correlation coefficient r value, and the lower left corner is the statistical test p-value. The above indicators were tested week by week in 11 ketotic cows and 10 CON healthy cows on the day of delivery and 1-7 weeks postpartum, (8 times monitoring).

**Figure 2:**
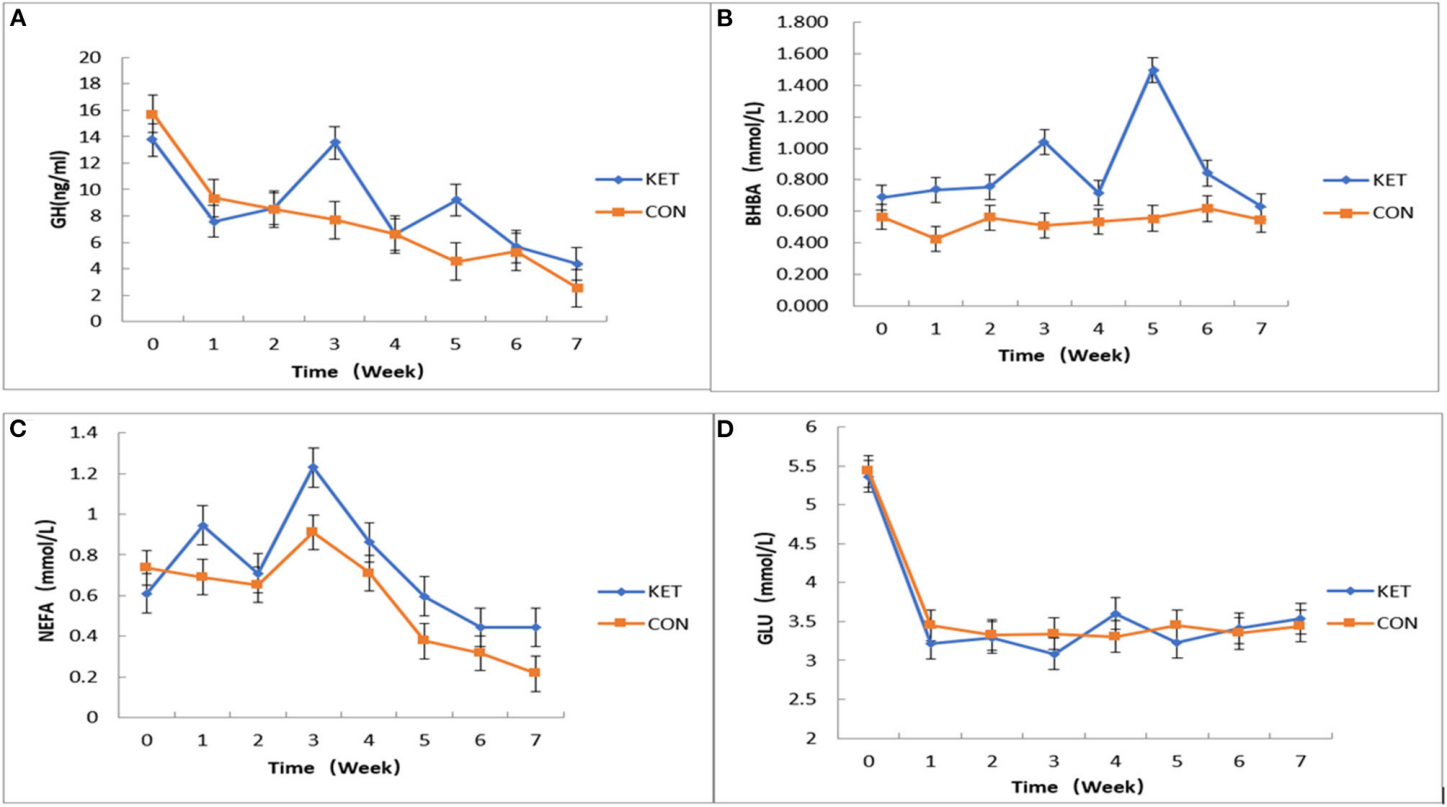
The effects of plasma levels of GH, BHBA, NEFA, and GLu on SCK and CON healthy dairy cows monitored weekly eight times. **(A)** The effect of plasma levels of GH on SCK and CON (*p* = 0.5512) healthy dairy cows during 0 to 7 weeks postpartum (*p* = 0.0416). **(B)** The effect of plasma levels of BHBA on SCK and CON (*p* = 0.7469) healthy dairy cows during 0 to 7 weeks postpartum (*p* = 0.0076). **(C)** The effect of plasma levels of NEFA on SCK and CON (*p* = 0.9519) healthy dairy cows during 0 to 7 weeks postpartum (*p* = 0.0047). **(D)** The effect of plasma levels of GLu on SCK and CON (*p* = 0.8082) healthy dairy cows during 0 to 7 weeks postpartum (*p* = 0.0001).

We applied JMP10 statistics (https://www.jmp.com/en_gb/support.html) to fit the mixed effect multiple regression model to measure the effect of plasma levels of GH, BHBA, NEFA and GLu related SCK indices on SCK-CON groups of dairy cows during 0 to 7 weeks postpartum testing time (Figure 2). Overall, the effect of plasma level of GH on the SCK and CON healthy dairy cows during 0 to 7 weeks of postpartum time showed a downward trend. However, the plasma level of GH in the third and fifth weeks of postpartum increased in the SCK group of dairy cows (Figure 2A). The effect of the plasma level of BHBA on the SCK and CON group was not significant. However, there were differences in plasma levels of BHBA in SCK and CON healthy dairy cows during 0 to 7 weeks of the postpartum time. Similar to GH, the plasma level of BHBA presented a higher concentration in the SCK group during the third and fifth weeks of the postpartum in the SCK group of dairy cows (Figure 2B). Overall, the effect of plasma level of NEFA content on the SCK and CON healthy dairy cows during 0 to 7 weeks of the postpartum time was not significant. However, similar to GH and BHBA, a higher difference in plasma level of BHBA during the third week of postpartum time was observed both in the SCK and CON health dairy cows. The peak value of FFAs was observed in the third week and then decreased gradually in the both SCK and CON groups (Figure 2C). Overall, the effect of plasma level of NEFA content on the SCK and CON group was not significant at 0–7 weeks postpartum. However, a higher difference of plasma level of BHBA during the fourth week of postpartum time was observed both in the SCK and CON health dairy cows. The peak value of FFAs in both SCK and CON groups were in the fourth week and then decreased gradually (Figure 2C). In general, the effect of the plasma level of GLu contents was not significant at 0-7 weeks postpartum. However, the plasma level of GLu was significantly lower in the first week than to zero weeks in both SCK and CON group, while, the GLu level was significantly higher in the fourth week in the SCK group (Figure 2D).

### Effects of LFT Indices in Ketotic and Healthy CON Dairy Cows During 0 to 7 Weeks of Postpartum

The results showed that GGT, TBIL, and DBIL were significantly higher in the ketotic cows than in healthy CON cows (*p* = 0.04, *p* = 0.005, and *p* = 0.009). However, the TP (*p* = 0.009) was significantly lower in the ketotic cows than that of healthy cows (Table 5).

**Table 5:** Comparison of LFT indices in ketotic and healthy CON dairy cows.

**Group**	**CON (*n* = 16)**	**KET (*n* = 16)**	***P*-value**
ALT, U/L	17.938 ± 1.304	20.375 ± 1.304	0.056
AST, logU/L	4.107 ± 0.259	4.151 ± 0.248	0.056
GGT, logU/L	7.3165 ± 1.149	7.7244 ± 1.1655	0.009^**^
TBIL, μmol/L	5.251 ± 0.773	8.688 ± 0.773	0.004^**^
DBIL, μmol/L	5.7133 ± 2.001	6.2047 ± 1.917	0.005^**^
ALB, g/L	30.937 ± 0.695	30.550 ± 0.695	0.696
TP, g/L	89.906 ± 1.921	85.200 ± 1.921	0.009^**^
GLOB (g/L)	58.969 ± 1.684	54.650 ± 1.684	0.079
A/G	0.530 ± 0.019	0.565 ± 0.019	0.209

^*^Denotes the difference is highly significant at p < 0.01.

**Figure 3:**
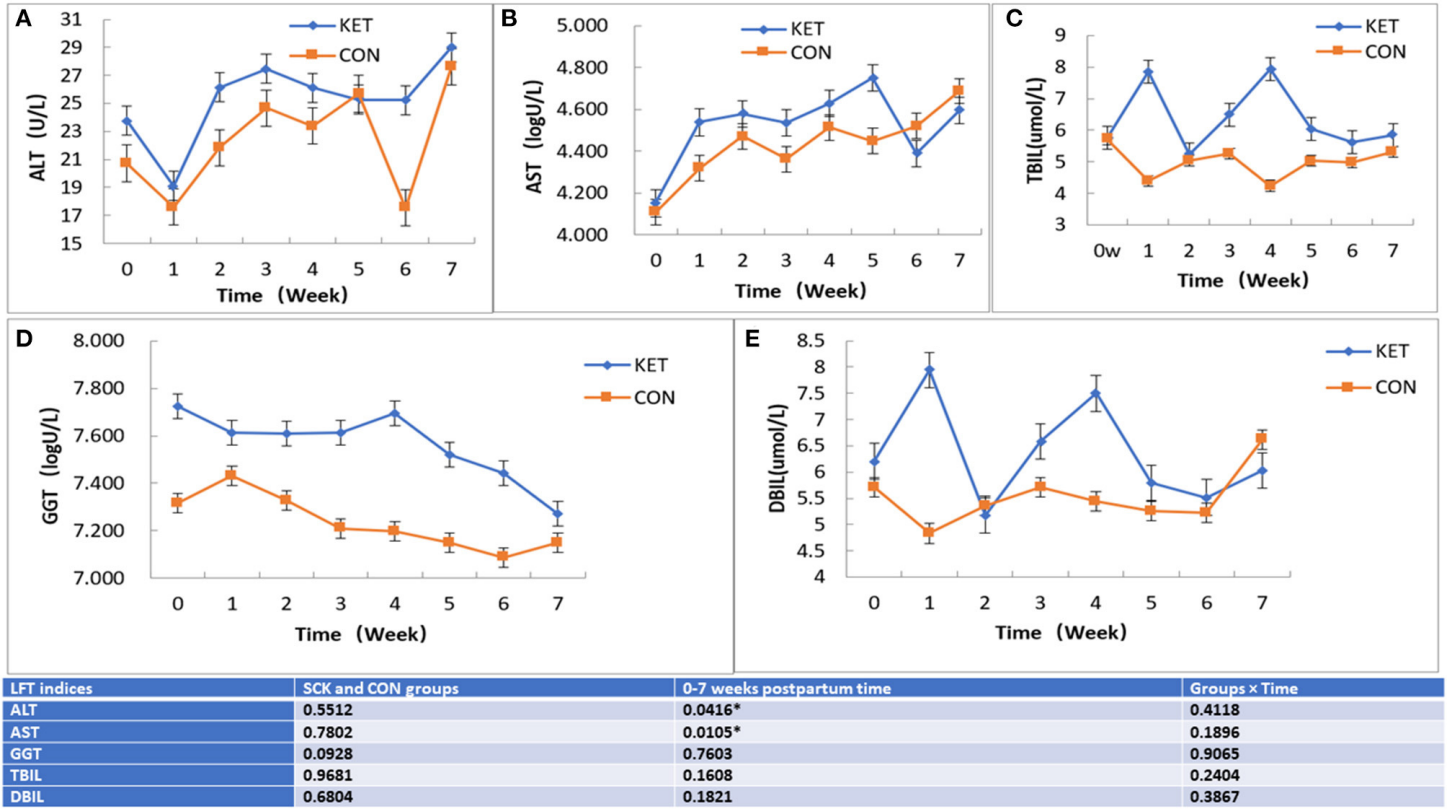
The effect on ALT, AST TBIL, GGT, and DBIL **(A-E)** LFT indices in SCK and CON healthy dairy cows monitored weekly eight times. **(A)** Effects of ALT-LFT in SCK and CON healthy dairy cows monitored weekly eight times. **(B)** Effects of AST-LFT in SCK and CON healthy dairy cows monitored weekly eight times. **(C)** Effects of GGT-LFT in SCK and CON healthy dairy cows monitored weekly eight times. **(D)** Effects of TBIL-LFT in SCK and CON healthy dairy cows monitored weekly eight times. **(E)** Effects of DBIL-LFT in SCK and CON healthy dairy cows monitored weekly eight times. *Denotes the difference is significant at *p* < 0.05.

The results showed that no significant differences between the SCK and CON groups for ALT-LFT. However, significant differences in ALT-LFT was observed during the 0 to 7 week postpartum time (Figure 3). In the case of the CON group, ALT decreased rapidly in the first week postpartum, then gradually increased and reached a peak in the fifth week and decreased again in the sixth week, while again increasing in the seventh week (Figure 3A). In the case of the SCK group, ALT decreased rapidly in the first week postpartum, increased in the second week, remained stable in the third to fifth weeks, followed by a sharp decrease in the sixth week and then a sharp increase in the seventh week. The ALT activity in the SCK group was higher than that of the CON group from delivery to the fourth week after parturition. In the CON group, there was a sharp decrease of ALT-LFT in the sixth week, but there was no difference in other weeks (Figure 3A).

Similar to ALT-LFT, no significant differences between the SCK and CON groups, and the significant differences during the 0 to 7 week postpartum time were observed for the AST-LFT. The AST in the CON group of dairy cows began to rise after delivery, and the AST level was relatively stable from the second to the sixth week after delivery and increased in the seventh week. In the SCK group, AST level rose sharply in the first weeks postpartum and was relatively stable in the next 3 weeks postpartum. Then, it increased again to the peak in the fifth week postpartum, then decreased sharply in the sixth week and increased sharply again in the seventh week. The AST level in the SCK group was higher than that of the CON group from zero to the fifth week postpartum, as shown in Figure 3B.

In the case of TBIL-LFT, no significant differences between the SCK and CON groups were observed during the 0 to 7 week postpartum time (Figure 3C). The CON group of TBIL-LFT decreased sharply in the first week after delivery and then remained at a relatively stable level during the second to the seventh week of postpartum. However, the TBIL-LFT level in the SCK group was increased in the first week, then a sharply decreased in the second week, and sharply increased in the third and fourth weeks, followed by a sharp decline in the fifth week and then remained stable in the sixth and the seventh weeks (Figure 3C). A contradictory trend for TBIL between SCK and CON groups was observed in the first and fourth weeks after parturition.

Similar to TBIL-LFT, no significant differences between the SCK and CON groups during the 0 to 7 week postpartum time were observed for the GGT-LFT (Figure 3D). The GGT-LFT level in the CON group increased in the first week after delivery and then substantially decreased from next week to the sixth week and slightly increase in seventh week. In the SCK group, GGT-LFT level also decreased in the first week, and then remained relatively stable in the next 3 weeks postpartum, and again increased in the fourth week, followed by a sharp decrease continuously in the fifth to the seventh week postpartum. Overall, the level of GGT-LFT in ketotic cows was higher than that of healthy CON cows (Figure 3D).

Similar to GGT-LFT and TBIL-LFT, no significant differences between the SCK and CON groups during the 0 to 7 week postpartum time were observed for the DBIL-LFT (Figure 3E). However, the DBIL-LFT level in the SCK group was much higher in comparison to the CON group in the first week after delivery, and in the fourth week postpartum. The DBIL-LFT level in the CON group decreased in the first week after delivery and then substantially increased in the next 2 weeks, and then stable the next 3 weeks, followed by a sharp increase in the seventh week postpartum. In the SCK group, the DBIL-LFT level sharply increased in the first week, and then sharply decreased in the second week, then again sharply increased in the third and fourth weeks, and sharp decreased in the fifth week, then remained relatively stable in the next 2 weeks postpartum. Overall, the level of DBIL-LFT in ketotic cows was higher than that of healthy CON cows (Figure 3E).

**Figure 4:**
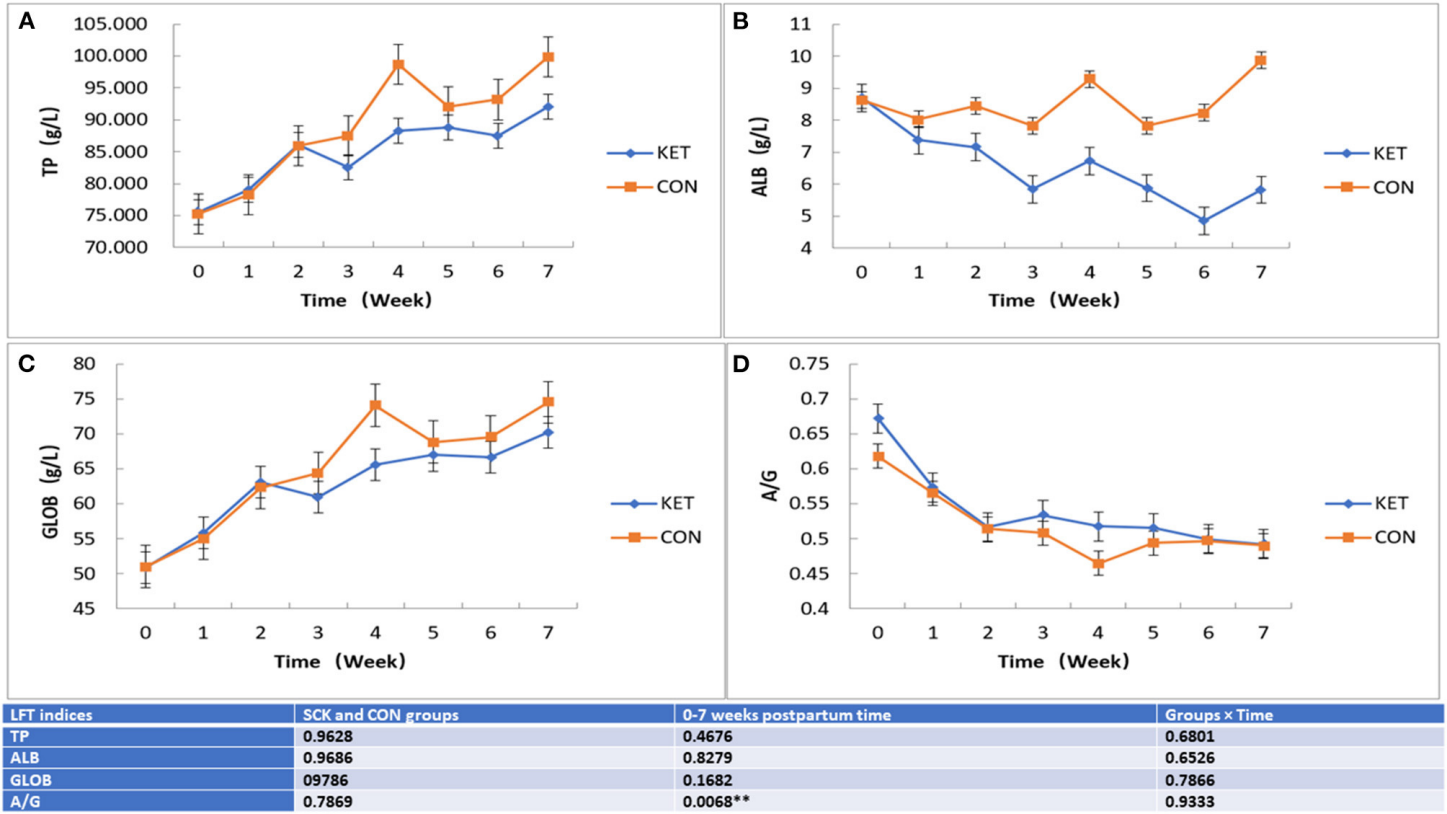
The effect on TP, ALB, GLOB, and A/G **(A-D)** LFT indices in SCK and CON healthy cows monitored weekly eight times. **(A)** Effects of TP-LFT in SCK and CON healthy dairy cows monitored weekly eight times. **(B)** Effects of ALB-LFT in SCK and CON healthy dairy cows monitored weekly eight times. **(C)** Effects of GLOB-LFT in SCK and CON healthy dairy cows monitored weekly eight times. **(D)** Effects of A/G-LFT in SCK and CON healthy dairy cows monitored weekly eight times. **Denotes the difference is highly significant at *p* < 0.01.

Overall, the effect on TP, ALB, GLOB and A/G LFT indices in ketotic and CON healthy cows showed that no significant differences between the SCK and CON groups. However, the differences in the levels of TP, ALB, GLOB and A/G LFT indices were observed during the 0 to 7 week postpartum time (Figure 4). In the case of TP and GLOB, an increasing trend of TP-LFT and GLOB-LFT levels was observed. Whereas in the case of ALB, a contrasting increasing and decreasing trend of ALB-LFT level were observed, and in the case of A/G a decreasing trend of A/G-LFT level was observed. In the CON group, the TP-LFT level gradually increased from the first week postpartum and reached a peak in the fourth week and decreased in the fifth week, then again increased in the sixth and seventh week postpartum (Figure 4A). In the case of the SCK group, TP increased sharply in the first and second weeks postpartum, then slightly decreased in the third week, followed by a slight increase up to the seventh week postpartum. In general, the TP activity in the SCK group was marginally higher than that of the CON group from delivery to the seventh week postpartum (Figure 4A). In the CON group, the ALB-LFT level was stable during 0 to 3 weeks, then increased in the fourth week, then decreased in the fifth week, followed by an again sharp increase in the sixth and seventh week postpartum (Figure 4B). In the case of the SCK group, ALB-LFT level decreased sharply from the first to the third week, then slightly increased in the fourth week, followed by an again decrease up to the sixth week, and finally increased in the seventh week postpartum (Figure 4B). Similar to TP-LFT, an increasing trend GLOB-LFT was observed in the SCK and CON groups. In the CON group, the GLOB-LFT level gradually increased from the first week postpartum to the fourth week and decreased in the fifth week, then stable in the sixth and finally increased in the seventh week postpartum (Figure 4C). In the case of the SCK group, TP increased sharply in the first and second weeks postpartum, then slightly decreased in the third week, followed by a slight increase up to the seventh week postpartum. In general, the TP activity in the SCK group was marginally higher than that of the CON group from delivery to the seventh week postpartum (Figure 4C). In the case of A/G, significant differences with decreasing trend A/G-LFT was observed in the SCK and CON groups (Figure 4D). Figure 3D shows that the A/G-LFT level in the SCK and CON groups was consistently decreased since delivery, and the cows in the CON group reached a low level in the fourth week, which then further increased and became stable. From the general trend, the A/G-LFT level of the SCK group was higher than the CON group from the day of calving to the fourth week after delivery (Figure 4D).

### The qRT-PCR Assays of GHR, JAK2-STAT5A, IGF-1, IGF-1R, PI3K-AKT, SREBP-1C, and PPARα Genes in Adipose Tissue of Dairy Cows During 0 to 7 Weeks of Postpartum

**Figure 5:**
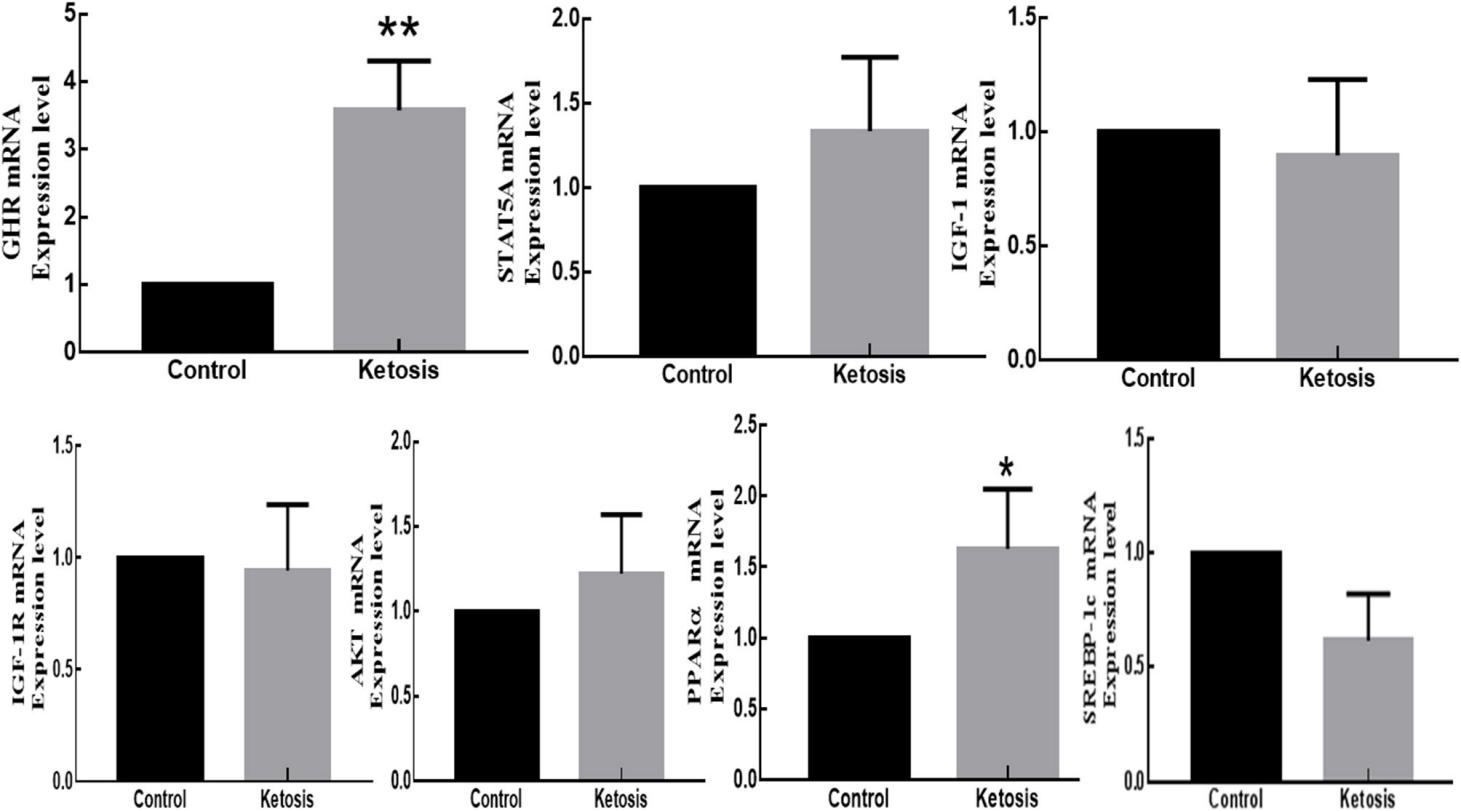
The mRNA expression of GHR, JAK2-STAT5A, IGF-1, IGF-1R, PI3K-AKT, SREBP-1C, and PPARα genes in adipose tissue of SCK and CON dairy healthy cows during 0 to 7 weeks of postpartum. *Denotes the difference is significant at *p* < 0.05; **denotes the difference is highly significant at *p* < 0.01.

According to the relative fluorescence quantitative PCR method, the mRNA expressions of GHR and PPARα genes in adipose tissue were significantly higher and upregulated in ketotic cows with *p*-values of 0.0034 and 0.0112, respectively. However, the mRNA expressions of STAT5A and PI3K-AKT genes in adipose tissue were marginally higher and upregulated in ketotic cows with *p*-values of 0.4867 and 0.1428, respectively. In contrast, the mRNA expressions of IGF-1, IGF-1R, and SREBP-1C genes in adipose tissue were higher and downregulated in healthy CON cows with *p*-values of 0.8963, 0.6107, and 0.0863, respectively (Figure 5).

## Discussion

### Effects of Plasma Levels of GH, BHBA, NEFA, and GLu in Ketotic and Healthy Dairy Cows During 0 to 7 Weeks of Postpartum

The dairy industry has achieved a marvelous pace in improving dairy cows' average milk production in the last two decades, primarily through genetic pool improvement with reproductive technology. However, genetic progress in feed efficiency has not progressed at the same rate as milk production. Therefore, high-producing dairy cows are mostly culled at a young age due to health issues such as hyperketonemia or SCK and other metabolic disorders (35). Normally, growth hormone stimulates protein, lipid and carbohydrate metabolism in both humans and animals. In some instances, the direct effect of growth hormone has been demonstrated, while in some cases IGF-I is thought to be the critical mediator. GH elevates the use of fat by stimulating triglyceride breakdown and oxidation in adipocytes. It has been reported that GH controls deviations in nutrient metabolism in the lactation period in high milk-producing dairy cattle (27). Thus, intense endocrine deviations occur throughout the transition phase to control the important metabolic changes in dairy cows (10). Previously, several reports revealed that shortly after parturition, the concentration of GH in blood surges, and this surge occurs together with a precipitous decline of IGF-I concentration in dairy cows' blood (28–30). In another study, it was reported that GH increases in blood encourage lipolysis in lactating cows' adipose tissue (27). In this study, the plasma levels of GH, BHBA and NEFA levels in SCK cows were significantly higher than CON cows, and the plasma level of GLu in SCK cows was significantly lower compared to CON cows during 0 to 7 weeks of postpartum. In general, the cow's appetite is considerably reduced in late pregnancy, and the birth causes a large physiological change in the body. Moreover, at the same time during the postpartum period, lactation causes a large loss of blood sugar and the body's energy intake is reduced, so the body cannot provide enough energy to meet the needs of itself and lactation. This leads to an NEB in the body, and fat mobilization may occur in the body under the action or participation of GH, which stimulates lipolysis *in vivo* as is evident by increased FAs (31) or glycerol (36) in the circulation after GH treatments in mammals. NEFA and BHBA are important indicators for determining the NEB (13). In this study, the plasma levels of NEFA and BHBA during the third week of the postpartum time in dairy cows. In a previous study, the plasma level of NEFA in postpartum cows is more significant than 0.7 mmol/L, indicating an NEB in the body (37). The average monitoring level of SCK in this experiment was 1.038 mmol/L, much higher than the energy negative equilibrium limit. The level of monitoring of NEFA shows that SCK cows' fat consumption is more serious. The recovery rate of NEB is lower than that of healthy cows (38). As SCK does not exhibit specific clinical symptoms, it is clinically diagnosed by detecting SCK indices indicators. Ketone bodies include β-hydroxybutyric acid, acetoacetic acid and acetone. The BHB has the highest stability; therefore, BHB concentration is used as a gold index for detecting SCK. In this study, the BHB of hyperketonemia or ketotic cows was significantly higher than that of CON cows. In SCK cows, BHB and NEFA were substantially higher than healthy cows (39). Blood GH is an essential indicator for determining the physiological function of the body. Many studies have shown that SCK cows will have pathological changes in high ketone bodies and hypoglycemia. The possible explanation is that cows in early lactation exhibit rapid adaptations to short-term exposure to energy balance such as feed deprivation by reducing energy losses (such as milk production) and by downregulating the production of satiety signals. Additionally, a high GH level promotes high lipid mobilization and an elevated hepatic removal of fatty acids and shifts the liver to stimulate ketone production. In consensus with that hyperketonemia, cows adapt rapidly by reducing milk and milk components' production. The sharp increase in plasma insulin, glucose or NEFA during early lactation could be a possible cause of hyperketonemia. It has been suggested that hormonal disparities could be a leading factor in SCK development besides negative energy imbalance (27). In this study, we found that GH concentration increased in the hyperketonemia group compared to the CON group which is in line with previously reported observations (37). In other studies, it was found that GH concentration increased during lactation in cows and ewes (22, 29, 30). Based on the above-mentioned evidence, we suggest that a high GH level may be an essential factor in promoting NEB and hyperketonemia in postpartum cows.

### Effects of LFT Indices in Ketotic and Healthy Dairy Cows During 0 to 7 Weeks of Postpartum

In postpartum dairy cows, the NEB causes adipose tissue mobilization which resulted in liver damage. Therefore, it is very important to monitor the occurrence of liver damage in perinatal dairy cows. The detection of liver-specific enzymes as LFT indices are often used as monitoring of liver damage. The investigated AST-LFT, ALT-LFT and GGT-LFT are closely related to liver metabolism and are commonly used LFT indexes to detect liver function. Our results showed that AST-LFT, ALT-LFT and GGT-LFT indices in plasma of ketotic cows and healthy cows had significant effects in ketotic cows, which indicated that there might be liver damage in ketotic cows. When liver cells are damaged, ALT and AST in the cells are released into the blood, resulting in the increase of enzyme activity in the blood. Our results findings are consistent with most of the previous studies which reported that the activity of AST and ALT in the blood of dairy cows with ketosis increased (40–43). In our study, the AST of healthy dairy cows increased from 0 weeks to 2 weeks postnatal, decreased in 3 weeks postnatal, and then continued to rise till the seventh week. The AST in the SCK group showed an upward trend after delivery and reached the peak value at 5 weeks after delivery, the overall average value was 86.893 u/l, which was lower than Yang's report (44). In the control healthy dairy cows, the plasma AST showed a trend of fluctuation and increase after delivery, gradually increased after delivery, decreased after 3 weeks, and continued to rise after delivery, the highest after seven weeks. According to Merck's Veterinary Manual, the normal reference range of AST (45.3-110.2 u/L) (45). In our study, nine cows were out of the normal range in 11 ketosis cows, while two cows were out of the normal range in 10 control cows, and the ratio of AST in ketosis cows out of the normal range is much higher than that in the control group. The peak of AST in ketosis cows was reached in 5 weeks postnatal, which was consistent with the change of BHBA. Because ALT and AST mainly reflect the damage of liver cells, AST, the exudative enzyme of liver cells, is usually used as a marker of liver fat deposition (46), it can be concluded that at this time, the damage of liver in ketosis cows is the most serious, and the abnormal change of liver function is the most prominent, but the average level of AST was higher (44). The ALT mainly exists in the cytoplasm of liver cells. When fatty liver occurs, the concentration of ALT in the plasma increases, but the low activity of ALT is not unique to the liver. The GGT is a kind of microsomal and membrane binding enzyme, which mainly exists in the liver, kidney and small intestine. The increase of GGT activity is related to the destruction of hepatocyte structure and bile duct disease. In our study, the GGT of healthy cows increased in the first week after delivery and then decreased gradually. In the SCK group, GGT decreased in 1 week postnatal, remained relatively stable in 1-4 weeks postnatal, and continued to decline in 5-7 weeks postnatal. In general, the GGT level of ketosis cows was higher than that of healthy cows. A study by MacMillan et al. reported that GGT has a low sensitivity index in the diagnosis of ketosis, so it is not suitable to be used as a sensitive index for the detection of ketosis (47). It may be that GGT is not a specific index for ketosis.

In postpartum dairy cows, one can expect the dynamic changes of serum bilirubin DBIL and IBIL LFT indices, which are products of the decomposition and destruction of aging red blood cells in the mononuclear phagocytic system of the liver, spleen and bone marrow in the blood circulation. The 80-85% of bilirubin in the blood comes from the decomposition of hemoglobin in the red blood cells. The bilirubin entering the blood combines with albumin and is transported to the liver for metabolism. After transformation by the liver cells, it is discharged into the capillary bile duct and discharged with bile. If the bilirubin produced in the body exceeds the capacity of the hepatocytes to process bilirubin, or the capacity of the hepatocytes to process bilirubin decreases, and the bilirubin removal obstacles can increase the bilirubin in the blood (48). Our results showed that TBIL and DBIL in the ketosis group were significantly higher than those in the control group. The study of Jia et al. (42) also found that TBIL and DBIL of ketosis cows were significantly higher than those of healthy cows. The dynamic analysis showed that TBIL and DBIL in the control group decreased rapidly after production, and gradually stabilized, and then increased again in the seventh week after delivery. In the ketosis group, the rise was rapid in the first week postnatal, then decreased in the second week postnatal, reached the peak in the fourth week postnatal, reached the bottom in the sixth week postnatal, and rose again. The overall trend is not stable, but it shows a downward trend of fluctuation, which is consistent with the research of Yang et al. (44). According to Merck Veterinary Manual, the normal reference range of normal DBIL in dairy cattle is 0-7.5umol/l, and the average value of the control group exceeds the normal range on the day of calving, indicating that the physiological change of calving causes the rise of DBIL in the liver (45). Ketotic cows were abnormally high except on the day of calving, 1 week and 4 weeks after delivery. The DBIL is a metabolite of red blood cells, which is metabolized into TBIL in the liver.

In postpartum dairy cows, one can expect the dynamic changes of plasma proteins TP and ALB LFT indices. The liver is the main site for the synthesis of TP and ALB, so the quality of the liver directly affects the synthesis of protein. The TP and ALB are important indicators to reflect the liver function, energy metabolism and immune function. When the liver function is impaired, the function of protein synthesis in the liver will be decreased, and the protein in the plasma will be reduced. The ALB is the most obvious decline, which can reflect the nutritional status of the body. While GLOB reflects the immune state of an animal body. In our study, TP of ketotic cows was significantly lower than that of healthy cows, and the ALB of ketotic and healthy cows had no significant difference, and the GLOB of ketotic and healthy cows had a statistically increased trend, and there were no significant differences between A/G groups. In a previous study, Jia et al. (42) found that TP and ALB in ketosis cows were lower than those in the control group (42). However, there was no significant difference in ALB between ketotic and healthy cows. In our study, the ALB of healthy cows was stable, and the ALB of ketotic cows was in a gradual decline in the sixth week. The dynamic trend chart of GLOB was similar to that of TP. The TP and GLOB of postpartum dairy cows increased gradually, but the relative health endurance of ketosis dairy cows increased slowly. The results showed that the concentration of white protein in ketosis cows was lower than that in the control group. The reason may be that the decrease in intake led to the decrease of amino acids absorbed by the digestive tract, which resulted in the shortage of raw materials for ALB synthesis. At the same time, TP, ALB, GLOB and other indicators of liver function were often associated with fatty degeneration in ketosis cows, which resulted in the decrease of liver synthesis function and the partial concentration of protein in the blood. The study of Jia et al. (42) reported that ALB of ketosis cows rose on the 14th day after delivery, and they concluded that the liver of ketotic cows was affected in different levels of degrees (49). In our study, the cows with ketosis rose in the 6th week after delivery. The reason for this difference can be explained that the incidence of ketosis cows in our study might be relatively late.

### Gene Expression qRT-PCR Assays of GHR, JAK2-STAT5A, IGF-1, IGF-1R, PI3K-AKT, SREBP-1C, and PPARα Genes in Adipose Tissue of Dairy Cows During 0 to 7 Weeks of Postpartum

The mRNA expression of GHRs typically exists on many tissue cell surfaces such as liver, adipose, kidney, and muscle. The GHRs consist of three domains, ectodomain, a transmembrane domain, and a cytoplasmic domain. The GH first binds to GHR on the cell surface and activates Janus kinase and other signaling factors signal transducers and transcription activators to stimulate metabolic growth and development. In this study, we found that the mRNA expression of GHR in adipose tissue was significantly higher and upregulated in hyperketonemia cows. Our results are in line with a previous study that found that an increase in blood GH promoted decomposition and lipolysis in adipose tissue (27, 38, 39, 50, 51). It has been suggested that lipolysis is closely related to the activation of STAT5. In this study, the expression of STAT5A mRNA in the hyperketonemia group's adipose tissue was not significantly different from that of healthy cows, however, the expression level was higher and upregulated in ketotic cows. A possible explanation is that STAT5A is a downstream molecule of the JAK2-STAT5A pathway, whose expression is affected by many factors, and the structure of the animal body is complex. In the liver, GH activates the JAK2-STAT5 pathway, increases the phosphorylation level of STAT5 and inhibits the activity of PPARα, a key enzyme of lipid oxidation, which in turn affects the expression of lipoxygenase genes and reduces lipid oxidation of hepatocytes. The PPARα is a ligand-activated nuclear transcription factor and a key transcription factor regulating the expression of hepatocyte lipid oxidase genes (ACO, CPT-1). In this study, the mRNA expression of PPARα in hyperketonemia cows was significantly higher and upregulated than in healthy cows. It can be speculated that the adipose tissue of ketotic cows is more prominent by PPARα-mediated regulation of adipocytic oxidative factors. Moreover, STAT5 is involved in GH-regulated lipolysis (52). After GH stimulates STAT5 activation in adipose tissue, phosphorylated STAT5 dimers are transferred into the nucleus to bind to DNA target sequences to regulate a series of fat synthesis genes (50–53). The PPARα is a downstream molecule of the JAK2-STAT5A pathway and its expression is inhibited in the liver. Previous studies reported that GH plays an essential role in adipose tissue, promotes its fat mobilization and breaks down fat into fatty acids (54). In this study, we found that GH might up-regulate PPARα through the JAK2-STAT5 pathway in adipose tissue and promote lipid oxidation. Moreover, GH induces activation of the JAK2-STAT5 pathway and increases the expression and secretion of mRNA for bovine hepatocyte IGF-1. The addition of BHBA or FFAs significantly inhibits GHR mRNA and JAK2 protein expression in the pathway and decreases the phosphorylation of STAT5 (55), which subsequently downregulates the expression and secretion of IGF-1 mRNA in hepatocytes. The BHBA impairs the intrahepatic GH-mediated JAK2-STAT5 pathway and downregulates the expression and secretion of IGF-1 in ketotic cows (56, 57). Although GH is classically regarded to work through JAK-STAT signaling, this is not always the case, especially in nutritionally restricted models. Fasting was shown to blunt JAK-STAT activation in human adipose and muscle tissues (32, 58). Similarly, GH-stimulated lipolysis in the adipose and muscle tissue of fasted human males was accompanied by the deactivation of STAT5B (32). In this study, the expressions of IGF-1, IGF-1R, AKT in adipose tissue in hyperketonemia cows were not significantly different from those in CON dairy cows. Through the PI3K-AKT pathway to regulate fat metabolism, the cow body is complex, and the differences between individuals are large, and the *in vivo* test is not sufficient to obtain accurate results. The SREBP-1c is a key transcription factor regulated by the PI3K-AKT pathway and it regulates lipid synthesis and transporter gene expression and is highly expressed in liver and adipose tissues (59), which regulates gene expression involved in adipogenesis by altering its mRNA levels (60). In this study, the expression of SREBP-1c in adipose tissue of hyperketonemia cows was low and downregulated in comparison to healthy cows, indicating that the expression of key transcription factors for fat synthesis in hyperketonemia cows was inhibited. This result is consistent with the fact that postpartum ketotic cows are thinner than healthy CON dairy cows.

## Conclusions

In the pathogenesis of hyperketonemia in dairy cows, significant and dynamic changes in the plasma levels of GH, BHBA, NEFA, GLu, and LFT indices were observed. The gene expression profile study in adipose tissues revealed the upregulation of GHR and PPARα genes in SCK affected cows in comparison to healthy cows. It can be speculated that somatotropin can act on adipocytes to promote lipolysis, but whether high plasma GH levels can directly cause hyperketonemia or its effect size, needs to be investigated. These findings provide valuable information that increases our understanding of the molecular mechanisms of hyperketonemia in dairy cows.

## Data Availability Statement

The original contributions presented in the study are included in the article/supplementary materials, further inquiries can be directed to the corresponding author/s.

## Ethics Statement

The animal study was reviewed and approved by College of Animal Science and Technology, Guangxi University under the supervision and protocol approved by the committee GXU-2017-026.

## Author Contributions

MM and BH designed the studies, write the original draft, data curation, formal analysis, funding acquisition, investigation, methodology, project administration, software, and prepared the manuscript with comments from HY, RH, PW, LG, YD, MA SS, MP, PS, KM, and CP including writing—review and editing of the manuscript. YH worked and prepared the revised manuscript with comments and corrections including writing review and editing. All authors have read and agreed to the published version of the manuscript.

## Funding

This project was supported by the National Natural Science Foundation of China (Nos: 573 31660697 and 31260631).

## Conflict of Interest

The authors declare that the research was conducted in the absence of any commercial or financial relationships that could be construed as a potential conflict of interest.

## Publisher's Note

All claims expressed in this article are solely those of the authors and do not necessarily represent those of their affiliated organizations, or those of the publisher, the editors and the reviewers. Any product that may be evaluated in this article, or claim that may be made by its manufacturer, is not guaranteed or endorsed by the publisher.
